# Oral administration of *Lactobacillus gasseri* SBT2055 is effective for preventing influenza in mice

**DOI:** 10.1038/srep04638

**Published:** 2014-04-10

**Authors:** Yosuke Nakayama, Tomohiro Moriya, Fumihiko Sakai, Noriko Ikeda, Takuya Shiozaki, Tomohiro Hosoya, Hisako Nakagawa, Tadaaki Miyazaki

**Affiliations:** 1 Department of Probiotics Immunology, Institute for Genetic Medicine, Hokkaido University, North-15, West-7, Kita-ku, Sapporo, 060-0815, Japan; 2 Milk Science Research Institute, Megmilk Snow Brand Co., Ltd, 1-2, Minamidai, 1-chome, Kawagoe, Saitama, 350-1165, Japan; 3 These authors contributed equally to this work.

## Abstract

The *Lactobacillus gasseri* SBT2055 (LG2055) is a probiotic lactic acid bacterium with properties such as bile tolerance and ability to improve the intestinal environment. In this study, we established that the oral administration of LG2055 exhibits efficacy to protect mice infected with the influenza virus A/PR8. The body weight losses were lower with the LG2055 administration after the PR8 virus infection. At 5 days after the infection, the virus titer was significantly decreased as was the amount of produced IL-6 in the lung tissue, the number of total cells in the bronchoalveolar lavage fluid was reduced by the LG2055 administration. The expression of the Mx1 and Oas1a genes, critical for the viral clearance in the lung tissues was increased by the pre-treatment with LG2055. These findings suggest that the LG2055 administration is effective for the protection against influenza A virus infection by the down-regulation of viral replication through the induction of antiviral genes expression.

Probiotics, live microorganisms have potential health benefits on the host, when administered in adequate amounts, they have been attracting growing interest for their health-promoting effects^1^, and have commonly been administered in fermented milk products.

The *Lactobacillus gasseri* SBT2055 (LG2055) is a probiotic lactic acid bacterium isolated from human feces with properties such as bile tolerance, ability to become established in the intestine and to improve the intestinal environment, and it has preventive effects on abdominal adiposity in rats and humans^2^,^3^,^4^,^5^. Therefore, this bacterium is chosen as a probiotic strain due to its suitability as a starter for preparing fermented milk. Further, with LG2055 it has been shown that its oral administration to mouse dams prevented rotavirus infection in their pups^6^.

Influenza A viruses are well known to cause highly contagious respiratory illnesses in humans and several animal species^7^,^8^. Influenza epidemics occur almost every winter, and the social and economic damage caused by severe influenza pandemics is an important issue in many countries. Aberrant production of inflammatory cytokines, such as tumor necrosis factor-α (TNF-α), interleukin-6 (IL-6), and interferon-γ (IFN-γ), is frequently observed in the course of lethal infections with influenza virus, and this is thought to be an important factor linked to viral pathogenicity^9^,^10^,^11^,^12^. In addition, influenza virus infections are quite dangerous to specific populations, such as pregnant women, diabetes patients, infants, and the elderly, who are known to be high risk groups^13^,^14^. Since these groups display some deficiency in immune responses, viral infections in these high risk groups frequently lead to severe and sometimes even lethal conditions. Therefore, maintaining the immune system in an appropriately robust condition is thought to be important for the prevention of the severe symptoms of influenza.

This study focuses on the immunomodulatory function of LG2055, and demonstrates that oral administration of LG2055 gives rise to increases in the survival rate of mice infected with the A/Puerto Rico/8/34 (PR8; H1N1) strain of the influenza virus. The oral administration of LG2055 protects the mice from a lethal PR8 virus infection, and the virus titer in the bronchoalveolar lavage (BAL) fluid is significantly decreased by LG2055 administration at 5 days after the virus infection. In addition, the oral administration of LG2055 induces the expression of the antiviral gene, myxovirus (influenza virus) resistance 1 (Mx1), and 2′-5′ oligoadenylate synthetase 1A (Oas1a) mRNAs in the lung tissues. These results indicate that the oral administration of LG2055 is efficient for the prevention of influenza by the inhibition of virus replication via up-regulation of the expression of antiviral genes such as Mx1.

## Results

### Oral administration of *Lactobacillus gasseri* SBT2055 (LG2055) increases the survival rate of mice after a lethal infection with influenza A virus

**Figure 1:**
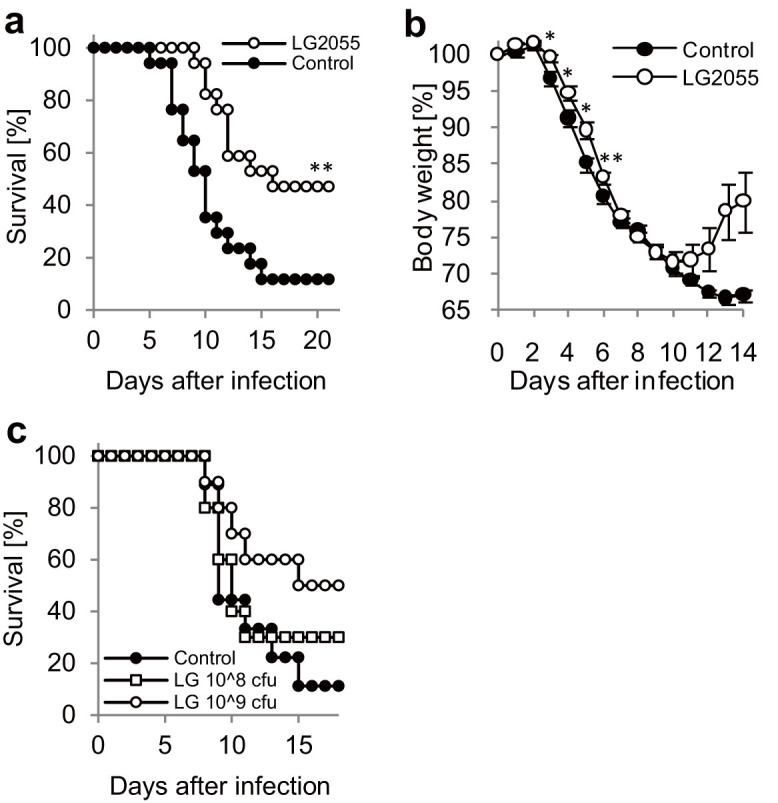
The oral administration of *Lactobacillus gasseri* SBT2055 (LG2055) protects mice from lethal PR8 virus infections. (a, b) The LG2055 (1.6 × 10^9^ cfu/mouse, n = 17), or 25% trehalose solution (n = 17) was orally administered to the C57BL/6N mice throughout the experiment once a day, from 21 days before the virus infection. After the administration for 21 days, mice were intranasally infected with the A/Puerto Rico/8/34 (PR8; H1N1) strain of influenza A virus at a titer of 1,000 pfu, and the survival rate (a) and body weight loss (b) of the mice were monitored. (c) All LG2055 groups were continuously orally administered at 1.0 × 10^9^ (n = 10) or 1.0 × 10^8^ (n = 10) cfu LG2055/mouse diluted in 25% trehalose solution during the experimental period. The control group was administered 25% trehalose solution (n = 9). After the administration for 21 days, the mice were infected with 50 μl of PR8 virus at a titer of 1,000 pfu. Data are presented as means ± SEM. *: p < 0.05. **: p < 0.01.

To investigate the effect of oral administrations of LG2055 on the prevention of influenza, we used the A/Puerto Rico/8/34 (PR8; H1N1) strain of the influenza virus, a laboratory strain which exhibits high virulence for C57BL/6N mice. The mice were orally administered with LG2055 or 25% trehalose solution once a day for 21 days, and subsequently were infected with a titer of 1,000 pfu of PR8 virus. Oral administration of LG2055 or 25% trehalose solution was continued once a day until day 20, and the body weight changes and clinical observations of the mice were monitored. The results show that the survival rate of the mice with the orally administered LG2055 was statistically significantly higher than that of the control mice (Figure 1a). As shown in figure 1b, the ratio of body weight losses was significantly lower in LG2055 administrated mice from day 3 to day 6. We also found the weight recovery by LG2055 administration from day 11 to day 14, but the difference did not reach statistical significance. In addition, the LG2055 solutions were administered at two concentration levels (1.0 × 10^9^ or 1.0 × 10^8^ cfu/200 μl), and the survival rates of the mice orally administered with these LG2055 solutions after the PR8 virus infection were monitored. These results indicated that the survival rate of mice tended to improve by the oral LG2055 administration in a dose-dependent manner, but the difference did not reach statistical significance (Figure 1c).

### Oral administration of LG2055 reduced PR8 virus replication in the lungs after infection

**Figure 2:**
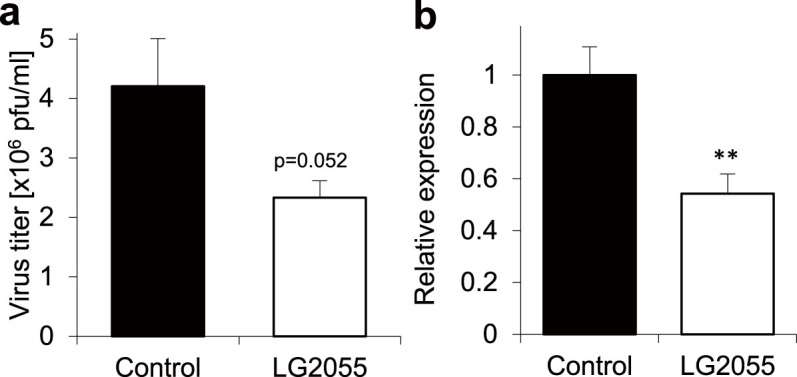
The oral administration of LG2055 inhibits influenza A virus replication in the lung. The LG2055 (1.0 × 10^9^ cfu/mouse), or 25% trehalose solution was orally administered to the C57BL/6N mice throughout the experiment once a day, from 7 days before the virus infection. After the 7 day pre-treatment, the mice were intranasally infected with the PR8 virus at a titer of 1,000 pfu. (a) The BAL fluids of the mice (n = 6 for each group) were collected at 5 days after infection. Virus titers in the BAL fluids were measured by plaque assays. (b) Total RNAs were isolated from the lung tissue of the mice infected with the PR8 virus at 5 days after infection. Subsequently, the RNAs were subjected to real-time PCR analysis using a specific primer set for PR8-NP. The data represent relative mRNA expressions which were normalized with the expression level of GAPDH mRNA. Data are presented as means ± SEM. **: p < 0.01.

The survival rate of mice was improved after 7 days with daily LG2055 administration, just like after 21 days with its daily administration (data not shown). Therefore, to investigate the effect of oral administration of LG2055 on the replication of the PR8 strain of influenza virus, the virus titers in the bronchoalveolar lavage (BAL) fluid by plaque assays were monitored using the mice with LG2055 administration for 7 days. As shown in Figure 2a, the results indicate that the virus titers in the BAL fluid tended to be lower in the LG2055-administered mice at 5 days after the PR8 infection. However, the difference did not reach statistical significance (p value = 0.052). To confirm the inhibitory effect on the viral replication by the LG2055 administration, real-time PCR was performed to analyze the expression level of the nucleoprotein (NP) gene of the PR8 virus in the lungs. As shown in Figure 2b, the expression of the PR8-NP gene was statistically significantly lower in the LG2055-administered mice at 5 days after the PR8 infection. These results demonstrate that oral administration of LG2055 reduces the viral replication in lungs of mice after infection.

### Oral administration of LG2055 reduced the inflammatory response in the lungs of mice after PR8 virus infection

**Figure 3:**
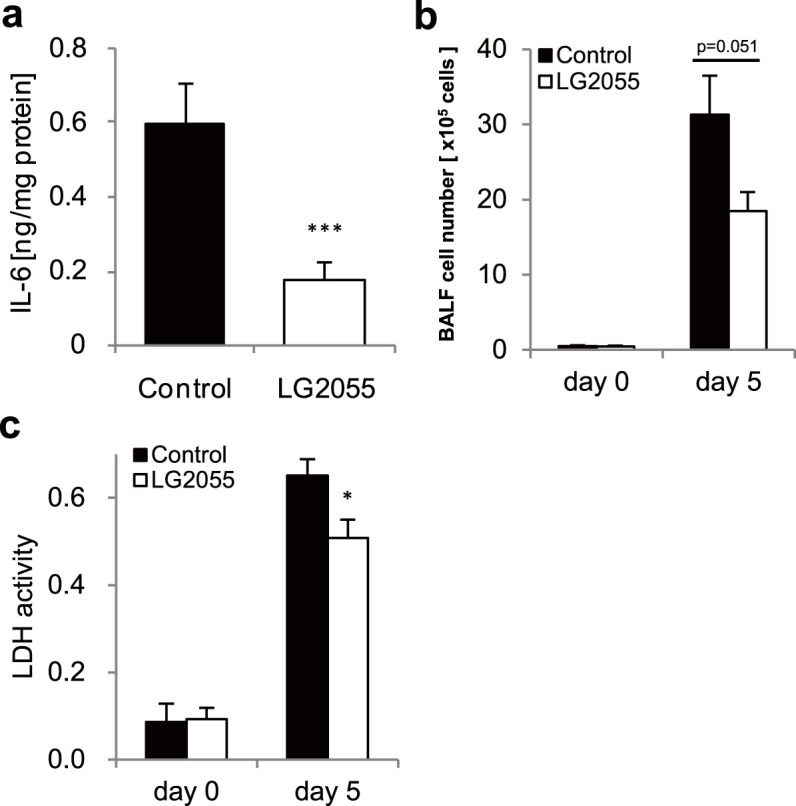
The oral administration of LG2055 impairs the inflammatory response to influenza virus infection. After the 7 day pre-treatment, the mice were infected with the PR8 virus at a titer of 1,000 pfu. (a) At 5 days after the infection, the lungs were removed and lung homogenates were assessed for IL-6 concentrations (n = 6/group). Data are presented as means ± SEM. (b) After the infection, the lungs were removed at the indicated time points and lung homogenates were assessed for LDH activity (n = 9/group at each time point). Data are presented as means ± SEM. (c) The BAL fluids were collected at the indicated time points and absolute numbers of BAL cells were counted using a hemocytometer. Data are presented as means ± SEM. *: p < 0.05. ***: p < 0.001.

To establish details of the mechanism for protecting the mice from a lethal titer infection of the influenza A virus by oral administration of LG2055, we estimated its effect to reduce the inflammatory responses in the lung of the mice after infection. Abnormal production of inflammatory cytokines is known to occur in the course of lethal influenza virus infections, and has been shown to be linked to disease severity^9^,^10^,^11^,^12^. At first, the expression level of a typical inflammatory cytokine, IL-6 in lung homogenates was evaluated using ELISA, and it was found that IL-6 production was significantly lower in LG2055-administered mice at 5 days after the PR8 infection (Figure 3a). In addition, as shown in Figure 3b, the results show that the number of BAL cells in LG2055-administered mice was lower than that in control mice at 5 days after the PR8 infection. Next, the activity of lactate dehydrogenase (LDH), one of the cytotoxic markers of inflammation in the BAL fluid was assessed using a Cytotoxicity Detection Kit (LDH). The LDH activity in the BAL fluid was significantly lower in LG2055-administered mice, compared with the control mice at 5 days after the PR8 infection (Figure 3c). These results suggest that the oral administration of LG2055 is effective to reduce the inflammatory response in the lungs of mice after PR8 virus infection.

### Expression of antiviral genes was induced in lung tissues by oral administration of LG2055

**Figure 4:**
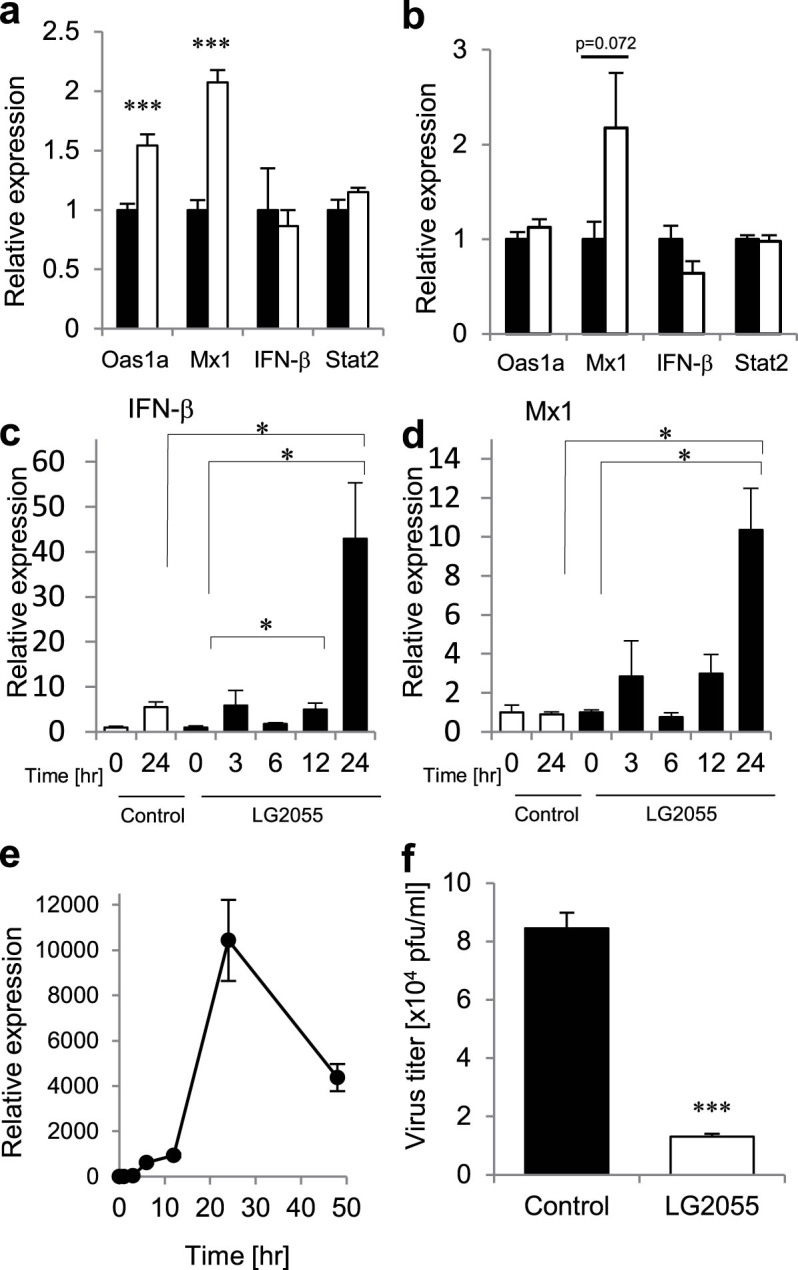
The oral administration of LG2055 has a potentiating effect that enables it to induce the antiviral genes in the lung tissues. (a, b) LG2055 (1.0 × 10^9^ cfu/mouse; open bars), or 25% trehalose solution (closed bars) was orally administered to C57BL/6N mice for 7 days. After the LG2055 pre-treatment, the gene expression of Mx1, Oas1a, IFN-β, and Stat2 in lung tissue (n = 9/group) (a) and alveolar macrophage (n = 9/group) (b) was assessed by real-time PCR. (c, d) RAW264.7 cells were stimulated with 100 μg/ml of LG2055 (closed bars) or PBS (open bars). At the each time point after stimulation, the cells were harvested, and the total RNAs isolated from the cells were subjected to the real-time PCR analysis using specific primer sets for IFN-β (c) and Mx1 (d) (n = 3/each time point). (e) RAW264.7 cells were infected with PR8 virus (MOI = 1), and then the cells were harvested at some time points of post-infection indicated in the figure. Total RNAs isolated from the cells were then analyzed by real-time RT-PCR using specific primer sets for PR8-NP. (f) RAW264.7 cells were stimulated with LG2055 (100 μg/ml) for 24 hrs, and then the PR8 virus was infected to the cells (MOI = 1). After the incubation, the virus titers in the cultured medium were measured by plaque assay. Data are presented as means ± SEM. *: p < 0.05. ***: p < 0.001.

Recently, it has been reported that the IFN response in macrophages isolated from the antibiotic-treated mice was impaired, resulting in low levels for the viral clearance after an influenza virus infection^15^. It was suggested that the commensal-derived signals modulate the responsiveness of macrophages to viral infection or IFN stimulation. To analyze the expression changes in antiviral genes such as Mx1, Oas1a, and Stat2 in the lung tissues by the oral administration of LG2055, real-time PCR analysis was performed. There was no difference in the expression levels of IFN-β and Stat2, however the expression level of Mx1 and Oas1a genes in the lung tissues was higher after the administration of LG2055 (Figure 4a). Therefore, the induction of Mx1 and Oas1a mRNA after LG2055 treatment is thought to be an important phenomenon to strengthen the protective activity from influenza A virus infection. Further, the increment of Mx1 mRNA expression was also observed in alveolar macrophages of mice after the oral administration of LG2055 (Figure 4b). In addition, we investigated the effect of treatment with LG2055 *in vitro* using cultured cells. Macrophage derived RAW264.7 cells^16^ were stimulated with LG2055, and the time-course for expression of IFN-β and Mx1 mRNA expression was analyzed by real-time RT-PCR. As shown in figure 4c, and 4d, the expression of IFN-β and Mx1 mRNA was significantly induced after 24 hrs with LG2055 treatment in RAW264.7 cells.

As shown in figure 4e, the expression of PR8-NP gene in RAW264.7 cells was increased and peaked at 24 hrs after the infection, but its expression in non-infected cells was not detected at any time (data not shown). Therefore, we confirmed whether LG2055 treatment influences the virus titer in RAW264.7 cells at 24 hrs after PR8 virus infection. Expectedly, the virus titer was decreased in the LG2055-treated cells compared to in untreated-cells. (Fig. 4f).

These results suggest that the LG2055 stimulation up-regulates Mx1 expression in macrophages and the administration of LG2055 is effective to reduce the virus titer in the lungs by the induction of expression of the antiviral gene, Mx1 and Oas1a in the lung tissues.

## Discussion

The present study demonstrates the protective effects of oral administration of *Lactobacillus gasseri* SBT2055 (LG2055) against influenza A virus infection. This effect enables mice to be resistant to a virus infection as shown by improvements in the survival rates (Figure 1) and by decrements in the virus titer in the lungs (Figure 2). It has been reported that massive inflammatory cell infiltration into the lungs and excessive proinflammatory cytokine production frequently occur in the course of influenza virus infections^17^,^18^. Therefore, we evaluated the number of inflammatory cells in the BAL fluid and the amount of produced IL-6 in the lung homogenates. As shown in Figure 3, the inflammatory cell infiltration and IL-6 production were inhibited in LG2055-administerd mice than in the control mice after the PR8 infection. This result suggests that LG2055 has a role in the preventive effect against the severe influenza via the regulation of these inflammatory responses.

Recent study has shown that the expression of genes associated with IFN activation and antiviral immunity was reduced in the alveolar macrophages of naïve antibiotic-treated mice, in which the number of intestinal commensal bacteria was decreased by the oral administration of broad-spectrum antibiotics^15^. It is known that the LG2055 is an intestinal commensal bacterium, and we examined whether LG2055 induces these genes (involved in the IFN signaling or antiviral immunity in the lung tissues). Our results suggest that the gene expression of Mx1 and Oas1a, which are critical for the antiviral responses by type I and type III IFNs was enhanced by the LG2055 administration for 7 days in the lung tissues and alveolar macrophages although the expression level of IFN-β was not higher after the LG2055 administration (Figure 4a, b). These results suggest that the effect of LG2055 might depend on the enhancement of host defense system before the virus infection. On the other hand, Mx1 and IFN-β mRNA were strongly induced in RAW264.7 cell after LG2055 treatment (Figure 4c, d). It should be pointed out that the inconsistency for IFN-β expression is still controversial. It is reasonable to assume that the intestinal cells were stimulated by bacterial components, and type-I IFN produced by these cells including macrophages in the intestine may secondarily stimulate the lung cells or macrophages in the lung for ISGs production. In addition, it has been reported that interferon-stimulated genes can also be activated through interferon-independent pathways^19^,^20^. Therefore, the expression of these antiviral genes by LG2055 administration may also be induced by IFN-independent pathways. Moreover, it has been demonstrated that oral administration of LG2055 to mouse dams prevented rotavirus infection in their pups via their breast milk enriched with rotavirus-specific immunoglobulin A (IgA)^6^. Therefore, LG2055 might have other protective effects against influenza virus infection such as enhancement of IgA production. Further investigation is required to better understand the detailed functions of LG2055 to enable the prevention of influenza.

## Methods

### *Lactobacillus gasseri* SBT2055 (LG2055) preparation and growth condition

LG2055 was provided from Milk Science Research Institute, Megmilk Snow Brand Co., Ltd. LG2055 was cultivated at 37°C in MRS broth (Difco, Detroit, Mich.) for 18 hours (1.5 × 10^9^ cfu/mL of medium) and harvested by centrifugation at 5000 × g for 10 minutes at 4°C. These harvested cells were washed twice with sterile PBS(−) and resuspended in 25% trehalose solution (5 × 10^9^ cfu/mL). Suspensions were stored at −80°C until used for experiments.

### Viral strain and mice

The A/Puerto Rico/8/34 (PR8; H1N1) strain of influenza A virus which was propagated in 10-day-old embryonated chicken eggs, was used for the experimental infection to mice. C57BL/6N mice (5–7 week old, male) were purchased from SLC Inc. (Shizuoka, Japan), were orally administered with the LG2055 (1.0 × 10^8^, 1.0 or 1.6 × 10^9^ cfu/mouse, 0.2 ml/mouse) or 25% trehalose solution through a syringe fitted with a ball-type feeding needle at once a day for 7–21 days, and subsequently were intranasally infected with the 1,000 pfu of PR8 strain of influenza A virus. All surgeries were performed under isoflurane anesthesia. Every effort was made to minimize suffering. Mice were lightly anesthetized with isoflurane (Dainippon Pharmaceutical, Osaka, Japan) and inoculated intranasally infected with the 1,000 pfu of PR8 strain on day 0. The survival rate and body weight were monitored daily until 21 days after the viral infection. Mice were euthanized by overdose of isoflurane on day 21 after the infection. After the infection, these mice were continued the administration with LG2055 at once a day. Mice that lost ≥40% of their original body weight and/or displayed evidence of pneumonia were euthanized by overdose of inhalant anesthetic. These experiments were conducted under animal BSL2 condition, and all animal experiments were performed in accordance with the guidelines of the Bioscience Committee of Hokkaido University and were approved by the Animal Care and Use Committee of Hokkaido University.

### BAL cell preparation

After the mice were sacrificed by isoflurane inhalation, the BAL fluids were collected in all experiments. BAL fluid cells were collected with three 1 ml aliquots of PBS containing 10 mM EDTA at 5 days after PR8 virus infection. The number of BAL cells was counted using a hemocytometer, and the supernatants of BAL fluid were used for plaque assay.

### Virus titration

To measure virus titers, the supernatants of BAL fluid were titered by plaque assay. Briefly, MDCK cells were infected with diluted the supernatants of BAL fluid at 37°C for 1 h. Cells were washed, overlaid with MEM containing 0.8% agarose and 0.0005% trypsin, and then incubated at 35°C for 48 h before counting plaques.

### Cell culture

A murine macrophage-like cell line, RAW264.7 (ATCC TIB-71)^16^ was cultured in RPMI-1640 medium (Sigma, St. Louis, MO) supplemented with 10% fetal bovine serum (FBS), 100 U/ml penicillin, and 100 mg/ml streptomycin, and was incubated at 37°C in 5% CO_2_.

### Challenge the virus to RAW264.7 cells

RAW264.7 cells were stimulated with LG2055 in a concentration of 100 μg/ml for 24 hrs, and then the PR8 virus was infected to the cells (MOI = 1). After the incubation for 24 hrs, the virus titers in the cultured medium were measured by plaque assay.

### Cytokine and lactate dehydrogenase (LDH) detection

Lungs were homogenized with tissue lysis/extraction reagent (Sigma-Aldrich) containing protease inhibitor mixture (Roche Diagnostics). Cytokine levels in the lung homogenates were determined using ELISA kits (BioLegend). For the detection of LDH activity in the BAL fluid, Cytotoxicity Detection Kit (LDH) (Roche Diagnostics) was used. The measurements were performed in accordance with the manufacturer's protocol.

### Real-time PCR analysis

Total RNA was extracted from the lungs using TRIzol reagent (Invitrogen). First-strand cDNA was synthesized from total RNA by using reverse transcriptase and random primers. Real-time PCR analysis was carried out using the KAPA SYBR Fast qPCR Kit (Kapa Biosystems). The primer sequences were as follows: PR8-NP, 5′-GATTGGTGGAATTGGACGAT-3′ and 5′-AGAGCACCATTCTCTCTATT-3′; IFN-β, 5′-TCCAGCACTGGGTGGAATGA-3′ and 5′-GGTACCTTTGCACCCTCCAG-3′; GAPDH, 5′-AAGGGCTCATGACCACAGTC-3′ and 5′-GGATGCAGGGATGATGTTCT-3′; Oas1a, 5′-GAAGAGGCTGATGTGTGGCT-3′ and 5′-TGTCCAGTTCTCTTCTACCTGC-3′; Stat2, 5′-AGAATGAGAAAGGAGGTGCTGG-3′ and 5′- AACTTTGCTCCAGCCGTCAA-3′; and Mx1, 5′-CCAACTGGAATCCTCCTGGAA-3′ and 5′-GCCGCACCTTCTCCTCATAG-3′.

### Statistical analysis

Two-tailed student's *t* test was used for statistical analysis. The Kaplan-Meier method with the log-rank test was used for analysis of mortality. P value of <0.05 was considered to be significant. The error bars indicate the standard error of the mean (S.E.M.).

## Author Contributions

Y.N., Tomohiro Moriya and Tadaaki Miyazaki designed research, Y.N., Tomohiro Moriya, F.S., N.I., T.S., T.H. and H.N. performed experimental work, Y.N., Tomohiro Moriya, F.S. and T.S. analyzed data, Y.N., Tomohiro Moriya and Tadaaki Miyazaki wrote the manuscript.
